# α-Glucosidase Inhibitory Activity of Tannat Grape Phenolic Extracts in Relation to Their Ripening Stages

**DOI:** 10.3390/biom10081088

**Published:** 2020-07-22

**Authors:** Auriane Dudoit, Nawel Benbouguerra, Tristan Richard, Ruth Hornedo-Ortega, Josep Valls-Fonayet, Gaëlle Coussot, Cédric Saucier

**Affiliations:** 1 SPO, Université de Montpellier, INRAE, Montpellier SupAgro, 34000 Montpellier, France; auriane.dudoit@umontpellier.fr (A.D.); nawel.benbouguerra@etu.umontpellier.fr (N.B.); 2 Molécules d’Intérêt Biologique, Unité de Recherche Œnologie, Institut des Sciences de la Vigne et du Vin (ISVV), Université de Bordeaux, EA 4577, USC 1366 INRA, 210 chemin de Leysotte, F-33882 Villenave d’Ornon, France; tristan.richard@u-bordeaux.fr (T.R.); ruth.hornedo-ortega@u-bordeaux.fr (R.H.-O.); josep.valls-fonayet@u-bordeaux.fr (J.V.-F.); 3 Institut des Biomolécules Max Mousseron (IBMM), Université de Montpellier, CNRS, ENSCM, 34000 Montpellier, France; gaelle.coussot@umontpellier.fr

**Keywords:** anti-diabetes, grape, ripening, α-glucosidase, phenolic compounds

## Abstract

The present study aimed to screen grape extracts as novel α-glucosidase inhibitors to prevent type-2 diabetes and hyperglycemia. The total polyphenol content (TPC) was measured by Folin-Ciocalteu assay and the stilbene, anthocyanin and flavan-3-ol compounds were measured by Ultra High-Performance Liquid Chromatography coupled to Mass Spectrometry (UHPLC-MS). The α-glucosidase inhibitory of seed and skin Tannat grape extracts at four ripening stages were investigated. The highest TPC values were measured in seeds at the “veraison stage” (65.29 ± 5.33 g of Gallic Acid Equivalent (GAE) per kilogram of Fresh Weight (FW)). This was in accordance with the high flavan-3-ol contents measured for these two extracts (43.22 ± 2.59 and 45.45 ± 6.48 g/kg of seeds FW, respectively). The skin and seed extracts at the first stage of ripening exerted strong α-glucosidase inhibition, exceeding 95% (*p* < 0.05). A high linear correlation (R = 0.723, *p* ≤ 0.05) was observed between flavan-3-ol contents and the α-glucosidase inhibitory activity. The stilbene contents and this activity were moderately to strongly anti-correlated (R = –0.828, *p* ≤ 0.05 for *trans*-resveratrol). The enzyme kinetic studies revealed a mixed type of inhibition. This study brings promising results for the therapeutic potential of seed and skin Tannat grape extracts as a functional food product with anti-diabetic activity.

## 1. Introduction

Four hundred and sixty-three million people worldwide suffer from diabetes, according to the International Diabetes Federation (IDF) [1]. IDF calls the phenomenon a true pandemic, as the progression is considerable. Predictions suggest that by 2030, 578 million people will have diabetes, and up to 700 million by 2040 [2]. Type-2 diabetes accounts for the majority of diabetes (90%) in the world. Type-2 diabetes, a prevalent endocrine-metabolic disorder, is the result of the body misusing insulin. It is characterized by hyperglycemia which is due to either paucity of insulin secretion by pancreatic β-cells or inefficiency of cells to use insulin against glucose. 

Twelve classes of medications, based on different mechanisms of action, are administered as monotherapy or in combination (i.e., metformin, sulphonylureas, glucagon-like peptide 1 analogues, Alpha-Glucosidase Inhibitors (AGI) in type-2 diabetes) [1,3]. Alpha-glucosidase, membrane-bound intestinal enzyme, is located in the epithelium of the small intestine. It hydrolyzes polysaccharides to *D*-glucose and other monosaccharides, which are then absorbed by the gut, and induces the postprandial hyperglycemia. It was reported that AGIs, oral antihyperglycemic drugs, attenuate postprandial blood glucose [3]. Acarbose is one of the most widely employed drugs for this treatment. It was associated with certain health benefits, such as the diminution in the risk of cardiovascular events. Nevertheless, despite its effectiveness, side effects such as gastrointestinal problems (i.e., diarrhea, nausea, flatulence) can severely limit its use. Therefore, it is interesting to look into the development of a new alternative treatment of natural origin to substitute these chemical compounds administered today.

Polyphenols, which are widely distributed in the plant world, are known for their numerous biological activities, such as antimicrobial, antioxidant, anticancer, anti-inflammatory, cardioprotective activities, or prevention of osteoporosis [4]. Among them, authors have been able to highlight polyphenols, such as stilbenes [5], cyanidin and its glycosides [6], anthocyanins [7,8], tannins [9] or chalcones, hydrocinnamic acids and isoflavones [10] as AGIs in type-2 diabetes pathology. 

**Figure 1:**
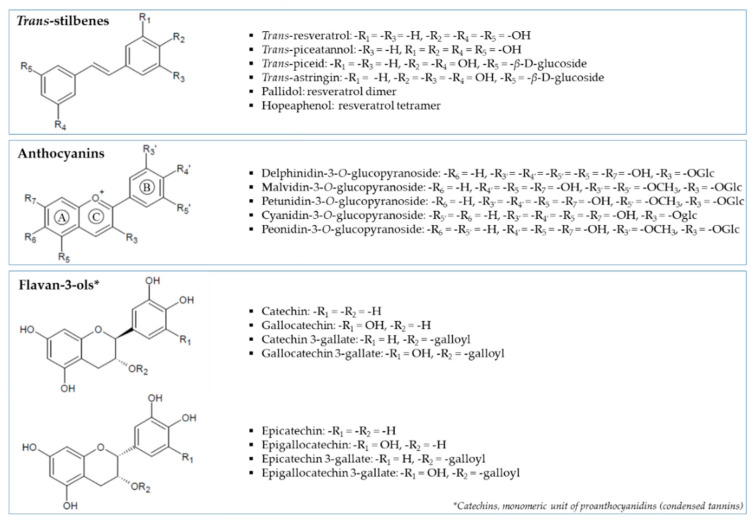
Structures of different polyphenol families and relative compounds identified in seed and skin grapes in the present work.

Grape (*Vitis vinifera* L.) is particularly rich in polyphenolic compounds, such as flavan-3-ols, catechins, anthocyanins and proanthocyanidins [11] (Figure 1). A few studies have demonstrated the inhibitory power of white wine grape pomaces (Chardonnay) against α-glucosidase, thus reducing postprandial hyperglycemia [12,13,14]. The main research has focused on red grape varieties (i.e., Cabernet Franc, Norton, Chambourcin) [14,15,16,17]. Kadouh et al. [16] found major efficiency for Tinta Cão, Syrah and Merlot extracts among six red wine grape pomace varieties on the α-glucosidase inhibitory potential in relation to higher total phenolic content (TPC). At equal or even lower concentrations, all the studied grape extracts demonstrated a superior efficiency compared to the widely prescribed AGI, acarbose. These findings are encouraging results but further investigations are needed to elucidate the complex composition-structure-activity relationships and maybe put on the drug market as a novel AGI. Sun et al. [18] showed, using bio-guided-Thin Layer Chromatography (TLC), that two stereoisomers of 6-O-*p*-trans-coumaroyl-*D*-glucopyranoside present in Tinto Cão grape pomace were potential α-glucosidase inhibitors. Other known compounds present in grape were also identified in other food matrices for their anti-postprandial hyperglycemic effect. Epigallocatechin gallate, present in tea extracts, has an important inhibition of α-glucosidase activity [18]. Two *trans*-resveratrol derivatives, rumexoid and piceatannol compounds, also possess some activities [5,19]. Zhang et al. [20] showed that anthocyanidins were more active than anthocyanins in the whole fruits of blueberry, honeysuckle and blackcurrant. The explanation of the anti-α-glucosidase activity by these two families of polyphenols in grape seed was proposed by Yilmazer-Musa et al. [18]. 

To our knowledge, only one publication was able to highlight the anti-postprandial hyperglycemic effect of skin grape pomaces for Tannat variety grape (origin: Montevideo, Uruguay) [21]. No study investigated the effect of the ripening stage of grapes on the α-glucosidase inhibitory activity of their extracts. The aim of the present study was then:

- To measure the stilbene, anthocyanin and flavan-3-ol content at the different ripening stages.
- To study the α-glucosidase inhibitory activity of seed and skin Tannat grape extracts at four ripening stages.
- To study the type of enzymatic inhibition by determining Lineweaver-Burk plots and kinetics constants calculations.

## 2. Materials and Methods 

### 2.1. Materials

#### 2.1.1. Chemicals and Reagents

Acetonitrile, sodium carbonate, hydrochloric acid, formic acid, ascorbic acid, gallic acid, Folin-Ciocalteu reagent, catechin, malvidine-3-*O*-glucoside, *trans*-piceid, *trans*-resveratrol, sodium phosphate dibasic dodecahydrate, potassium phosphate monobasic, sodium carbonate and dimethyl sulfoxide (DMSO) were purchased from Sigma-Aldrich (Saint-Quentin Fallavier, France). Sodium acetate and trifluoroacetic acid were obtained from Carlo Erba Reagents (Peypin, France), and methanol and phloroglucinol from Biosolve Chimie (Dieuze, France). The solvents used were High Performance Liquid Chromatography (HPLC) grade. Deionized water was obtained from a Milli-Q Advantage A10 purification system from Millipore (Fontenay sous Bois, France). Alpha-glucosidase from *Saccharomyces cerevisiae*, acarbose and *para*-nitrophenyl alpha-D-glucopyranoside (*p*-NPG) were purchased from Analytic Lab (Castelnau-le-Lez, France). *Trans*-astringin and *trans*-piceatannol were obtained from Carbosynth (Compton, UK) and ChromaDex (Irvine, CA, USA), respectively. Hopeaphenol, isohopeaphenol, Ɛ-viniferin, δ-viniferin and ω-viniferin were isolated from a grapevine raw shoot. The *cis*-isomers were obtained using Ultraviolet-C (UV-C) irradiation (254 nm) from *trans*-isomers.

#### 2.1.2. Fruit Materials

Tannat grapes were harvested at different stages of ripening on the 28 June (first stage), 11 July (before veraison), 25 July (veraison) and 14 September 2017 (maturity) from the INRAe experimental vineyard (Montpellier, France) (Coordinates: 43°37′02.7″ N 3°51′22.3″ E, average annual precipitation: 629 mm, average annual temperature: 15.85 °C, and soil: gravels and river sand). The whole grapes were stored at –80 °C in plastic bags until polyphenol extraction.

### 2.2. Methods

#### 2.2.1. Polyphenol Extraction

Skins and seeds of thirty Tannat grapes at different stages of ripening were manually separated from the pulp. Biological replicates consist of samples issued from random sampling in the vineyard that were processed separately (three times thirty Tannat berries) to provide a better representation of biological variance across samples for different stages of ripening. A total of ninety samples were collected per ripening stages.

The total polyphenols were extracted from seeds and skins with 100 mL of acetone/water (70/30: *v*/*v*) then put under deoxygenation with nitrogen for 5 min to minimize the natural oxidation. After stirring for 18 h in the dark, the solution was filtered through a 0.45 µm filter paper then evaporated under vacuum pump V-100 (Suction capacity (1.5 m^3^/h, final vacuum (10 mbar)) at 37 °C [22]. The resulting products were freeze-dried between 48 and 72 h under a pressure < 10 Pa and stored at –20 °C until their uses in analytical and enzymatic assays. Skins and seeds were weighted before extraction (Fresh Weight = FW). 

#### 2.2.2. Ultra High Performance Liquid Chromatography-Mass Spectrometry (UHPLC-MS) Analysis of Stilbenes

The stilbene analysis was performed by Ultra-High-Performance Liquid Chromatography-Mass Spectrometry (UHPLC-MS) based on a previous method [23]. The freeze-dried skin samples and stilbene standards were solubilized in methanol/water (1/1: *v*/*v*) at an appropriate concentration (20 g/L).

The system was composed of an UHPLC system (Agilent Technology 1260 Infinity, Agilent Technologies, Santa Clara, CA, United States), hyphenated to an Agilent Technologies 6430 Triple Quadrupole Detector. An Agilent Poroshell 120 EC-C_18_ column (150 × 2.1 mm, 2.7 µm particle size) was used as a stationary phase. The mobile phases consisted of 0.1% formic acid in water as solvent A and 0.1% formic acid in acetonitrile as solvent B, using the following gradient: 5–17.5% B (0–5 min), 17.5–33% B (5–7.5 min), 33% B (7.5–10 min), 33–40% B (10–15 min), 40–95% B (15–16 min), 95% B (16–19 min) and 5% B (19–21 min). The flow rate was fixed at 0.3 mL/min and the column temperature was set at 35 °C. The injection volume was 4 µL. The detection of stilbenes was in the Multiple Reaction Monitoring (MRM) mode with specific transitions for each compound, and for quantification purposes, a calibration curve was built in the range of 0.05–26 mg/L with pure stilbenes standards. All tests were carried out in triplicate and the results were expressed as milligram per kilogram of skin grapes (FW).

#### 2.2.3. UPLC-Photodiode Array (PDA)Analysis of Anthocyanins 

The total anthocyanin content was measured using UPLC-PDA, as described by Pérez-Magariño et al. [24] and Giuffrè et al. [25], with some modifications. The freeze-dried skin samples were solubilized in methanol/water (80/20: *v*/*v*) at an appropriate concentration. 

The UPLC system was a Waters Acquity (Saint-Quentin-en-Yvelines, France), with a photodiode array detector (PDA), LC pump and an auto sampler. The column used was a reversed phase UPLC with an Acquity UPLC BEH C_18_ column (2.1 × 50 mm, 1.7 μm particle size) (Saint-Quentin-en-Yvelines, France). The temperature of the column was 50 °C. The method used a binary gradient with mobile phases containing 1% *v*/*v* aqueous trifluoroacetic acid (mobile phase A) and acetonitrile (mobile phase B). The 40 min elution method at flow 0.25 mL/min was 0 min 1% B, 5 min 8.8% B, 30 min 20.6% B, 30.5 min 96% B, 34 min 96% B, 34.1 min 1% B and 40 min 1% B. The detection was monitored at 520 nm. Apparatus was controlled by Empower™ 3 acquisition software (Waters, Saint-Quentin-en-Yvelines, France). A calibration curve of malvidine-3-*O*-glucoside was used. All tests were carried out in triplicate and the results were expressed as gram of Malvidine-3-*O*-Glucoside Equivalent (MGE) per kilogram of skin grapes (FW).

#### 2.2.4. UPLC-PDA Analysis of Flavan-3-ols

Total flavan-3-ols assay using UPLC-PDA was performed as described by Kennedy et al. [26]. Briefly, a solution of 0.1 N HCl in MeOH, containing 50 g/L phloroglucinol and 10 g/L ascorbic acid, was prepared. The freeze-dried skin and seed grapes were dissolved and reacted in this solution at 50 °C for 20 min, and then combined with 5 volumes of 40 mM aqueous sodium acetate to stop the reaction. 

The chromatographic apparatus conditions were the same as described in experimental Section 2.2.3. The temperature of the column was set at 40 °C. The method used a binary gradient with mobile phases containing 1% *v*/*v* aqueous trifluoroacetic acid (mobile phase A) and acetonitrile (mobile phase B). The 20 min elution method at flow 0.45 mL/min was 0 min 2% B, 8 min 6% B, 14 min 20% B, 14.1 min 99% B, 16 min 99% B, 16.1 min 2% B and 20 min 2% B. Eluting peaks were monitored at 280 nm. A calibration curve of catechin was used. All tests were carried out in triplicate and the results were expressed as gram per kilogram of skin or seed grapes (FW).

#### 2.2.5. TPC Determination

The TPC was estimated by the Folin-Ciocalteu colorimetric method [27]. The freeze-dried skin or seed samples were solubilized in methanol (1/200: *w/v*). 20 μL of the diluted extract, 1.58 mL of water, and 100 μL of Folin-Ciocalteu reagent were mixed. After 30 s, 300 μL of 20% sodium carbonate solution (*w*/*v*) was added. The reaction mixture was thoroughly shaken and left for 2 h in the dark at room temperature (20 °C). Then, the specific absorbance was measured at 700 nm against the blank prepared for each series of determination in such a way that the sample was replaced by the methanol, using an Agilent Cary 60 Ultraviolet-Visible (UV-Vis) spectrophotometer (Santa Clara, CA, United States). The calibration curve of gallic acid was used to determine the concentration of polyphenol in samples. All tests were carried out in triplicate and the results were expressed as gram of Gallic Acid Equivalent (GAE) per kilogram of FW.

#### 2.2.6. α-Glucosidase Inhibition Assays

##### Set-up of the α-Glucosidase Inhibition Assays in Microplate Format

To test the inhibitory effect of studied skin and seed Tannat grapes harvested at different stages of ripeness, the protocol used was inspired by Pramod et al. [28] based on the use of *p*-NPG, a colorless, unnatural substrate for α-glycosidases. Optimization of reactant volumes, concentrations, pH and incubation times was carried out. The optimized protocol is as follows: 10 µL of freeze-dried grape (Inhibitor) was dissolved in DMSO at 0.4 g/L, then mixed with 60 µL of phosphate buffer solution (100 mM, pH 6.8) containing 10 µL of α-glucosidase enzyme (1 U/mL) into a native polystyrene microwell (Nunc MaxiSorp^TM^, VWR, Fontenay-sous-Bois, France). The Enzyme/Inhibitor (E/I) mixture was subjected to a moderate linear shaking for 15 min at 37 °C in a Heidolph incubator 1000 equipped with a Titramax 1000 shaker (Heidolph Instruments GmbH & Co. KG, Schwabach, Germany). After pre-incubation, 20 μL of a 2.5 mM *p*-NPG (Substrate, S) solution in distilled water was added into each well. The mixture was incubated again for 20 min at 37 °C with shaking. The enzymatic reaction was stopped by adding 50 µL of 0.1 M Na_2_CO_3_ solution to reach pH 10. At this pH, the resulting hydrolysis product (*para*-nitrophenol, *p*-NP) is under its basic form (yellow color). The 4-nitrophenolate form has a maximum of absorbance at 405 nm. All absorbance measurements were recorded at this wavelength with an Infinite 200^TM^ absorbance microplate reader from Tecan (Lyon, France). This instrument has a measurement range up to 3.6 absorbance units. All experiments were repeated at least four times (*n* ≥ 4) per analytical condition. Acarbose, a synthetic AGI, was prepared in distilled water at 0.4 g/L and was used as a positive control for inhibition. Negative control was conducted by replacing the grape extracts with DMSO. No inhibition occurs in that case; thus, maximum absorbance values are obtained with negative controls. Background signal called “blanks” was obtained by substituting the α-glucosidase enzyme with phosphate buffer solution (100 mM, pH 6.8). The blank value was 0.060 ± 0.004 (*n* = 16). Performance criteria of the optimized protocol (working range, precision and selectivity) were evaluated according to the European Medicine Agency guideline for the validation of bioanalytical methods [29]. Precision of an assay is usually expressed as the standard deviation (SD) or relative standard deviation (RSD) or coefficient of variation CV (the standard deviation divided by the mean value), reported as a percentage. For precision parameters, within-day RSD is noticed as CV_R_. Intermediate precision (also called within-laboratory) is expressed as CV_RW_ being carried out by changing operators, conducted on different days, using daily prepared reagent solutions, and different Tannat grape extracts. CV_RW_ includes biological and technical variabilities.

##### Data Interpretation

All data are reported as the mean ± the standard deviation (SD) from at least four replicate experiments. The α-glucosidase inhibitory activity was expressed as percentage inhibition by using the following formula:

(1) $$\%\;\mathrm{inhibition}=\frac{A\left(\mathrm{control}\right)-A\left(\mathrm{sample}\right)}{A\left(\mathrm{control}\right)}\;\times \;100$$

where A_(sample)_ is the measured absorbance at 405 nm obtained with grape extracts and A_(control)_ corresponds to the absorbance of the negative control. Both samples are prepared simultaneously. 

##### Application of the Microplate Assays to Kinetic Studies of α-Glucosidase Inhibition

The inhibition modes of α-glucosidase by skin and seed Tannat grape extracts at the first stage of ripening were determined by using Lineweaver-Burk plots. The Michaelis-Menten constant (K_m_) and the maximum reaction velocity (V_max_) were determined following the optimized protocol described previously. The experiments were carried out with increasing concentrations of α-glucosidase solution (0.025, 0.050, 0.075 and 0.100 g/L) and *p*-NPG (2.5, 3.0, 3.5, 4.0 and 4.5 mM). The absorbance readings for enzymatic kinetic were started just after the addition of the substrate solution. The released *p*-NP was monitored at 405 nm every minute for 50 min. All absorbance values were included in the working range of the above optimized procedure. All inhibition assays were carried out in triplicate.

#### 2.2.7. Statistical Analysis

The total polyphenolic content and the α-glucosidase inhibitory effects of grape skin or seed extracts were tested by an analysis of variance (ANOVA) on all the data. A Tukey test was carried out and where *p* < 0.05 was considered as significant. Pearson’s correlation coefficient (R) and stepwise linear regression were carried out for the determination of correlations between the dependent variable (α-glucosidase inhibitory activity) and the four independent parameters (polyphenolic families, stilbene, anthocyanin and flavan-3-ol contents). The *p*-values less than a confidence level of 95% *(p* ≤ 0.05) were considered as statistically significant. All these analyses were performed using XLSTAT software (Addinsoft version 19.01, Paris, France).

## 3. Results and Discussion

### 3.1. Evolution of Phenolic Composition in Tannat Grape during Maturation

The total phenolic composition (TPC, stilbenes, flavan-3-ols, anthocyanins) from skin and seed Tannat grape extracts during ripening were measured and are detailed in Table 1 and Table 2. 

#### 3.1.1. TPC Analysis 

The TPC from the freeze-dried skins and seeds at different ripening stages were determined, as described in Table 1. Skin and seed grapes was the major source of polyphenol compounds with an abundance in seeds, and the same finding was noted by Xu et al. [30]. In seeds, the highest TPC values were attained at the before veraison and veraison, compared with other stages. In skins, the TPC values were found to be higher in the first stage and maturity stage. Obreque-Slier et al. [31] showed the same evolution of TPC compared to our study. The TPC declined from 2.2 to 1.1 mg GAE/g in skins, and it increased from 21.8 to 22.5 mg GAE/g then declined to 16.6 mg GAE/g in seeds. This variability in content might be due to the methods and solvents used to extract the phenolic compounds [32] or the environmental stress, variety, agronomical practices, geographic locations, maturity, irrigation and plant pathogen presence [33,34]. The decline of TPC may be explained by the partial oxidation of polyphenol compounds [35]. The content and the phenolic composition differ considerably between seeds and skins and between the different stages of ripening, which will probably act on the biological activity linked to the inhibition of the enzyme α-glucosidase.

#### 3.1.2. Total Stilbene Content

Stilbenes were found only in skins with a content that varied with the ripening stages. The total stilbene content increased from the first ripening stage to before veraison. At veraison, the content was about 1.08 ± 0.08 mg/kg of skins, which then increased significantly to 5.18 ± 2.04 mg/kg of skins at maturity. Gil-Muñoz et al. [36] have studied the stilbene content of two grape varieties at harvest and have measured levels at 32.50 and 33.67 mg/kg of skin on grapes harvested in 2014, and 19.03 and 31.43 mg/kg of skin on grapes harvested in 2015 for the Monastrell and Tempranillo varieties, respectively. 

As for total stilbenes, the concentrations of most individual stilbene compounds increased during ripening (Table 2). The *trans*-piceid presented the highest content at all ripening stages, similar to other grapes (Monastrell and Tempranillo at harvest) that accumulate resveratrol in their glucosylated form, i.e., *trans*-piceid at 10.29 and 9.34 mg/kg skin, respectively [36]. *Trans*- and *cis*-resveratrol were found at lower levels. Our results are in accordance with Gatto et al. [37], who studied the content of resveratrol in 78 *Vitis vinifera* varieties during ripening and had the same evolution. They found that resveratrol content raised from 0.01 and 0.4 to 0.03 and 1.7 mg of resveratrol per kg of grape in the two lowest producing varieties. The same trend was also observed for other grape varieties, such as Pinot Noir, Merlot or Cabernet Sauvignon [38,39]. Stilbenes’ synthesis and accumulation was in direct relation with several biotic and abiotic factors, mainly ultraviolet radiation [40], that may explain the variability on stilbenes content found in the literature. 

The accumulation of stilbenes from veraison to maturity was in relation to the increases of stilbene synthase, phenylalanine ammonialyase and 4-coumarate-CoA ligase expression, responsible for the stilbenes’ synthesis and accumulation [37]. The stilbene content is influenced by several factors such as varieties, years and viticultural conditions [36]. The climatic conditions of the region act considerably on the stilbenoid compounds content of grapes; it was already shown that in dry and warm climates, the stilbene content is low, whereas it is higher in regions associated with harsher climates [41].

#### 3.1.3. Total Flavan-3-ol Content 

The most abundant phenol compounds found in grapes were the flavan-3-ols with a higher content in seeds compared with skins at all ripening stages (Table 1), which is in accordance with previous results [11,42]. In skins, the highest flavan-3-ol content was measured at the first ripening stage and before veraison, then decreased significantly at veraison and maturity. In seeds, the highest content was before veraison and at veraison. Lorrain et al. [43] studied the phenolic composition of two red grape (skin and seed) varieties, Merlot and Cabernet Sauvignon, from veraison to maturity. A similar evolution was observed, with a difference in the content that could be due to the method to extract polyphenols. According to Adams [44], the decline during ripening may be associated to the natural oxidation of flavan-3-ol compounds. 

The contents of extractable proanthocyanidins are reported in Table 2. Their content in Tannat skins were lower than in seeds, in agreement with Obreque-Slier et al. [45]. In skins, only catechin was identified as a terminal unit, while epicatechin-phloroglucinol and epigallocatechin-phloroglucinol were identified as an extension unit with an abundance of epicatechin-phloroglucinol, which is in agreement with Downey et al. [46]. The catechin and epicatechin-phloroglucinol contents decreased significantly from the first stage to veraison. Oppositely, epigallocatechin-phloroglucinol increased and it was absent at veraison and maturity. Obreque-Slier et al. [45] have demonstrated that in skins of both Carménère and Cabernet Sauvignon, catechin was identified as a terminal unit that coincided with our study, whereas epigallocatechin-phloroglucinol, epicatechin-phloroglucinol, catechin-phloroglucinol and epicatechin-3-*O*-gallate-phloroglucinol were identified as extension proanthocyanidin units. The absence of these compounds in our study may be due to a variety effect. In seeds, catechin, epicatechin-phloroglucinol and epicatechin gallate were found as a terminal unit, whereas catechin-phloroglucinol, epicatechin-phloroglucinol and epicatechin-3-*O*-gallate-phloroglucinol were detected as extension units, with an abundance of epicatechin-phloroglucinol content, which is in agreement with Obreque-Slier et al. [45], Downey et al. [46] and Kennedy et al. [47]. The decline of all individual flavan-3-ols after veraison was explained by some partial oxidation phenomenon [47]. 

#### 3.1.4. Total Anthocyanin Content

The anthocyanins are the colored pigment of skins and were found to be absent in seeds, in accordance with Tkacz et al. [48]. They are also absent in the green stages (first stage and before veraison) of grape skins, in agreement with a previous study [49]. The synthesis started at veraison and accumulated during ripening (Table 1). Our study is in agreement with Boido et al. [50] who studied the total anthocyanins content of Tannat grapes during ripening. They found that the content increased from 1807 mg/kg of grape to 3810 mg/kg of Tannat grapes. The same trend was also observed for other grape varieties, such as Nerello, prunesta, Merlot or Cabernet Sauvignon [25,43].

Nine individual anthocyanins were determined in skin Tannat grapes from veraison to maturity and are reported in Table 2. The content of all individual compounds increased from veraison to maturity with an abundance of malvidin-3-*O*-glucoside, in accordance with Mulero et al. [34]. Lorrain et al. [43] showed that malvidin-3-*O*-glucoside was the major anthocyanin compound in Cabernet Sauvignon (7.39 mg MGE/g of Dry Weight (DW) skin) and Merlot (5.17 mg MGE/g DW skin). The variability observed in the content may be due to the content of primary metabolites, especially sugars, that play a major role in anthocyanins biosynthesis [51] and may also be due to the genetic control of varieties studied [52]. 

Sugars’ accumulation during ripening can play a major role in the synthesis of anthocyanin compounds, it was considered as a substrate for anthocyanins formation and as a regulator in the synthesis [53]. The anthocyanin contents were mostly affected by altitude and environmental factors, such as temperature, that stimulate regulatory genes’ expression and varieties. It has been found that the higher temperatures, such as 35 °C, favor the anthocyanin degradation, however, the lower temperatures, around 25 °C, induce the anthocyanins synthesis [54]. 

### 3.2. Optimization and Performances of α-Glucosidase Inhibition Microplate Assays 

A microplate-based assay for α-glucosidase activity measurement was developed for rapidly determining inhibition effects of seeds or skins Tannat grapes on α-glucosidase (EC 3.2.1.20) activity. This enzyme hydrolyzes (1→4)-linked α-D-glucose terminal residue of mono- and poly-saccharides. *p*-NPG, a colorless, unnatural substrate for α-glucosidases, was used as an indicator of enzyme activity since its hydrolysis product is *p*-NP. The absorption spectrum of the basic form of *p*-NP displays a maximum at 405 nm. Reactant concentrations and volumes, pH and incubation times were optimized for control α-glucosidase activity. The whole protocol enables 96 samples to be studied from a single measurement, minimizing operator handlings errors. The effect on the α-glucosidase activity of the final content of DMSO (6.5% *v*/*v*) per well was also checked. The optimized protocol is detailed in the Experimental Section. The assay detection mixture was performed in a final volume per well of 140 µL, and contained 10 mU of α-glucosidase, 4 µg of inhibitor (grape extracts) and 50 nmoles of the *p*-NPG substrate. The performances of the proposed optimized protocol were controlled by studying its working range and its precision.

The working range of the assay was determined by the measurement of seed extract (maturity stage) calibration standards, prepared as described in the Experimental Section, within the range 0.025 to 1.00 g/L (*n* = 4 replicates per concentration). This experiment was reiterated over three independent days (*n* = 12 data per concentration level). This gave similar inhibition binding curves over the 3 days (data not shown) with good precision (Table 3). In addition, blank samples revealed that there was no interference during the sample analysis. Maximum absorbance values were obtained with negative controls reported as 0 g/L. In Table 3, we can see this A_(control)_ value corresponded to 2.50 ± 0.10 absorbance, which confirmed that using 10 mU of α-glucosidase permitted to obtain homogeneous results from day to day. This corresponds to the upper limit of the working range, meaning (100%) of the active enzyme. Consequently, the half-maximum absorbance obtained in the experimental working range was 1.25 ± 0.10. This value corresponded to 50% inhibition of α-glucosidase activity. The working range is limited by a concentration of extracts to be no more than 0.5 g/L. Indeed, as reported in Table 3, at 1.0 g/L of inhibitory extracts, variability of the inter-day assays dramatically increased up to about 46.8%, probably because the reaction medium became hazy due to possible insoluble components at this concentration. Thus, the interaction between grape extract and α-glucosidase was not favorable. Based on these results, all the following studies were done with grape extracts prepared at a concentration less than 0.5 g/L. 

Precision of the assay was evaluated with within-day (repeatability) and inter-day (intermediate precision) experiments. The precision parameters ((within day RSD noticed as CV_R_) and intermediate precision RSD (CV_RW_)) were estimated for negative control and 3 levels of grape extract (0.25 g/L, 0.50 g/L and 1.00 g/L) of extracts solution added to the wells. Repeatability was evaluated by repeating the assays on the same microplate (8 replicates) during the same day using the same solutions. Statistically identical results (*p* > 0.05) were found for all wells. The effect of random events on the precision of the assay was studied by changing factors (3 days, 3 operators and freshly prepared reagents). ANOVA was used to estimate the precision of the assays and calculations with a minimum of 24 replicates performed. Table 3 gives precision results with CV_R_ at 3.5% and CV_RW_ at 8.8% for the negative control. For extract samples prepared at a concentration lower than or equal to 0.5 g/L, CV_R_ are less than 11.5% and CV_RW_ ranged from 12.0% to 26.1%. The results were satisfactory for biological products due to the number of analytical steps (extraction, storage with freeze-drying and thaw laboratory preparation, enzyme). The assay fulfilled intermediate precision. So, to directly estimate percent of inhibition of the α-glucosidase activity after being in contact with Tannat grape extract, the formula (1) was applied. The effects of all seed and skin extracts at all stages of ripening were studied.

### 3.3. α-Glucosidase Inhibitory Activity of Grape Extracts

**Figure 2:**
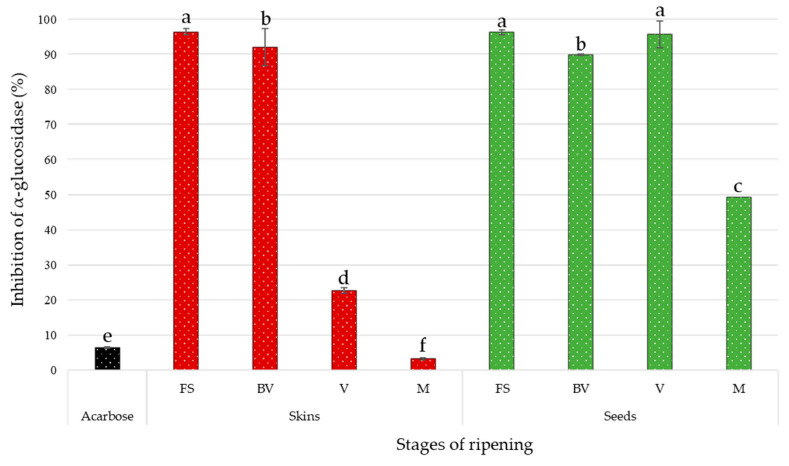
Inhibitory effects of Tannat skin and seed extracts (0.4 g/L) and positive control (acarbose at 0.4 g/L) on α-glucosidase (pH 6.8, T = 37 °C) at different stages of ripening: First Stage (FS), Before Veraison (BV), Veraison (V) and Maturity (M). Values are means of four replicates. Significant differences between treatments at *p* < 0.05 are indicated with letters as measured by Tukey test.

The obtained results, expressed in percentage of α-glucosidase inhibition, are illustrated in Figure 2. Positive control with acarbose prepared at 0.4 g/L was carried out simultaneously. The inhibition percentage for this synthetic AGI was 6.46 ± 0.22.

At the concentration of 0.4 g/L of dry extract, all the extracts showed an inhibition on the enzymatic activity of α-glucosidase. Except for the skin extract at maturity stage, all these natural extracts proved to be more effective against α-glucosidase compared to the acarbose, a commercial AGI. Overall, the inhibitory effects of skin extracts decrease throughout the different stages of ripening, losing almost all of its activity. As for seed extracts, the inhibitory power remains almost constant during the first three stages of ripening and ends up losing almost 50% of its activity at maturity. The first stage of skin extract and seed extracts, first stage and veraison, exerted the strongest inhibition of α-glucosidase. Both seed and skin extracts, at the same stage of ripening (before veraison), have also demonstrated their efficiency. The inhibitory power of these two extracts is nevertheless statistically different from those reported previously. The seed extract at maturity and skin extract at veraison also exhibited significant activity, like inhibition of α-glucosidase. Finally, the skin extract at maturity showed the least activity compared to the other extracts. Its inhibitory potential was less than the chemical control tested at the same concentration. These two extracts were significantly different.

Fernández-Fernández et al. [21] have observed the potential antidiabetic of hydro-alcoholic-acid extract of Tannat grape skin. As opposed to the results obtained in our study, acarbose demonstrated better efficacy than the extracts. For the same variety grape, phenolic composition could be affected by several factors, such as sunlight [54], altitude, climate [54,55], grape ripeness and soil conditions [56]. Nevertheless, the α-glucosidase inhibitory data reported in this manuscript were in accordance with previous studies. Grape extracts have been shown to be potent inhibitors of the enzyme compared to acarbose [12,14,15,16,17]. As reported by Hogan et al. [14], the inhibitory effect (63.9 and 42.4%) of the red and white wine grape pomaces (Cabernet Franc and Chardonnay) at 10 µg/mL surpassed that of acarbose (150 µg/mL), which exerted 26.5% inhibition. Also, Zhang et al. [16] showed significant inhibition of red Norton grapes of yeast α-glucosidase at a concentration range of 14.3–285.7 µg/mL compared to commercial AGI (285.7 µg/mL). The observed variability of the inhibitory activity may be due to varietal differences and differences in sample preparation conditions.

**Figure 3:**
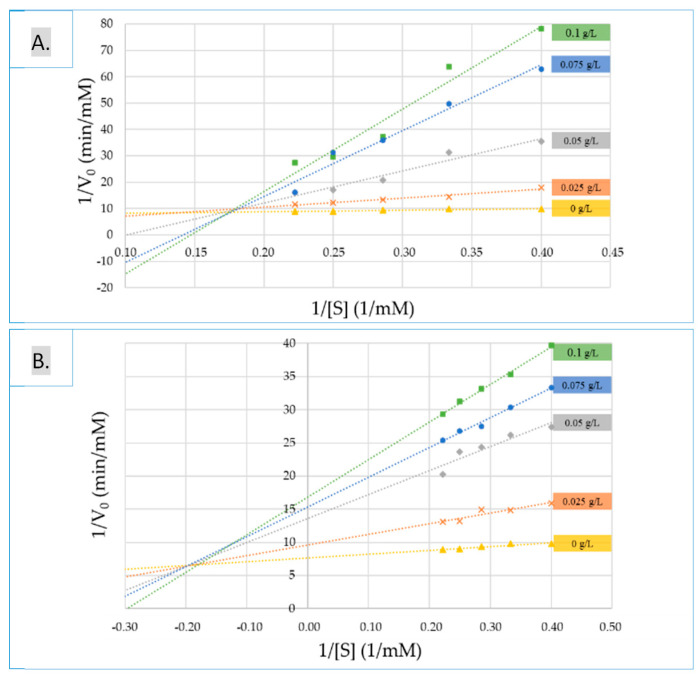
Lineweaver-Burk double reciprocal plots of (**A**) skin and (**B**) seed extracts at the first stage of ripening for the α-glucosidase activity mode.

In order to get further, the mode of action of two of the most active extracts (seed and skin extracts at the first stage of veraison) was investigated. To find the inhibition mechanism against α-glucosidase, inhibitory kinetics were analyzed by Lineweaver-Burk plots and are presented in Figure 3. The reciprocal velocity (1/v) versus the increasing substrate concentration was plotted. α-glucosidase presented a Michaelis-Menten constant (K_m_) of 0.7395 mM/L for *p*-NPG and a maximum reaction velocity (V_max_) value of 0.1301 1/min (Table 4). Apparent V_max_ values with the increasing concentrations of seed extracts (0.025, 0.050, 0.075 and 0.100 g/L) decrease, and the K_m_ values raise. In the presence of increasing concentrations of skin inhibitor, V_max_ values were found to be 0.2657, 0.0823, 0.0283 and 0.0218 1/min and K_m_ values of 9.0839, 10.0296, 7.0813 and 6.8055 mM/L, respectively. The results revealed mixed inhibition mode for both extracts.

### 3.4. Correlation between Chemical Composition and α-Glucosidase Inhibitory Activity

**Figure 4:**
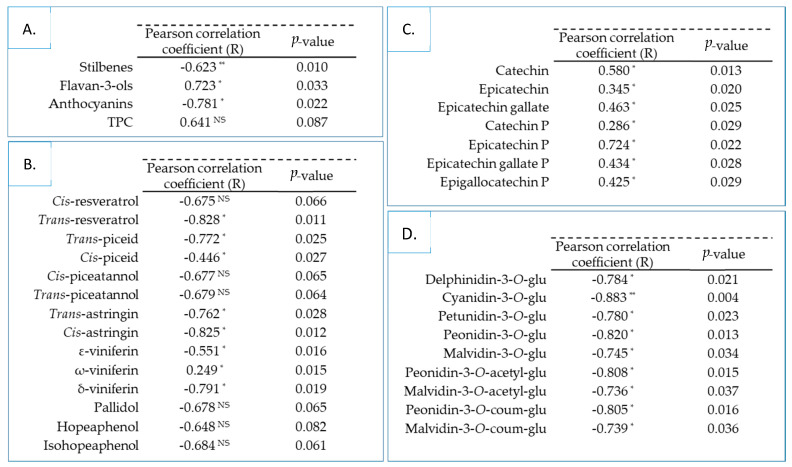
Pearson’s correlation coefficient (with α = 0.05) between α-glucosidase inhibitory activity of all skin and seed Tannat grape extracts and total polyphenol families (**A**), stilbene (**B**), flavan-3-ol (**C**) and anthocyanin (**D**) compounds. * Indicates significance at *p* ≤ 0.05, ** Indicates significance at *p* ≤ 0.01. NS = Non-Significant, TPC = Total Polyphenol Content, glu = glucoside, coum = coumaroyl, P = Phloroglucinol.

Previous results were used to assess the relationship between total polyphenol contents, individual stilbene, flavan-3-ol, anthocyanin compounds and the potential antidiabetic of skin and seed Tannat grape extracts. The Pearson’s correlation coefficients (R) and the probability *p*-values were calculated and are detailed in Figure 4.

In our study, the Pearson correlation test showed that α-glucosidase inhibitory activity was negatively correlated with stilbenes and anthocyanins (Figure 4A). Regarding the TPC, we obtained a positive but non-significant correlation, depending on the desired α-level with the enzymatic inhibition in contrast to flavan-3-ols, which had a significantly positive correlation coefficient.

Additionally, stilbenes were moderately to strongly anti-correlated, with the exception of ω-viniferin, which was weakly positively correlated (Figure 4B). Although Kerem et al. [5] and Zhang et al. [19] demonstrated that *trans*-resveratrol had an inhibitory activity on α-glucosidase, our data did not support this observation. In our study, the skin Tannat extract at the first stage of ripening exhibited a significantly lower content of *trans*-resveratrol (Table 2) with greater inhibitory activity (Figure 2) than the same extract at veraison or maturity stages. The overall contents of stilbenes in skin grape extracts at the first stage and veraison were significantly identical (Table 1). Nevertheless, the latter had shown inhibitory activity with three times less activity. Zhang et al. [19] was able to highlight the common structural characteristic, among 32 stilbenes, presenting the best enzymatic efficacy: the presence of C_4′_-OH.

The correlation analysis showed that individual anthocyanins were strongly negatively correlated with the potential antidiabetic of grape extracts (Figure 4D). Cyanidin-3-*O*-glucoside presented the strongest negative correlation with a Pearson coefficient of –0.883 (*p* ≤ 0.01). These findings suggested that the strong anti-correlation observed for anthocyanins was directly related to the fact that this family was only present in the two samples (skin grapes at veraison and maturity) (Table 1) with the least activity (Figure 2).

The anthocyanin aglycones as well as cyanidin compounds revealed a much stronger antidiabetic activity than its glycoside form, such as cyanidin-3-*O*-galactoside or cyanidin-3,5-diglucoside [6,8,57]. It suggested that anthocyanins were potent inhibitors of intestinal sucrose after being hydrolyzed in intestine. Furthermore, sugar units linked to anthocyanins played an essential role in exerting biological activity, as described by Akkarachiyasit et al. [6]. The replacement of 3-*O*-galactose by 3-*O*-glucose moiety (diastereoisomer/hydroxyl group on C_4_-position) of cyanidin revealed less power. The substitution of a disacharide (rutinose) instead of a monosaccharide of 3-*O*-cyanidin fraction might also be a significant factor in the activity [58]. Sun et al. [17] hypothesized that a hydroxyl substituent at the C_1_-position of saccharide moiety in phenolic glycosides, such as the chemical structure of acarbose, would induce the potency of intestinal sucrose inhibition.

Finally, significant positive correlations among all individual flavan-3-ols were observed (Figure 4C). The results indicated that the epicatechin-phloroglucinol and catechin compounds have the strongest correlations, and weak correlation with catechin-phloroglucinol. As a matter of fact, the epicatechin-phloroglucinol content of the two least active extracts was significantly lower than the most active ones (Table 2). The importance of this correlation resides in the fact that this compound has been predominantly identified in all the Tannat grape extracts. Matsui et al. [59] reported that the α-glucosidase inhibitory activity of monomeric flavan-3-ols (catechins) were determined as (-)-epigallocatechin-3-*O*-gallate > (-)-epicatechin-3-*O*-gallate > (-)-epicatechin > (-)-epigallocatechin > catechin. This study also illustrated the importance of stereoisomers of catechins on activity. Catechins, with a 2,3-*trans* structure (catechin-3-*O*-gallate and gallocatechin-3-*O*-gallate), showed less activity than epicatechins that have 2,3-*cis* structure ((-)-epicatechin-3-*O*-gallate and (-)-epigallocatechin-3-*O*-gallate). In addition, the galloylated monomers had higher inhibition than non-galloylated. These structural observations of stereoisomerism aspect on α-glucosidase inhibitory activity were also observed by Gamberucci et al. [60].

These observations do not mean that the presence or absence of certain compounds would explain all the activity. Presumptively, the mentioned activities might arise due to synergistic interaction between individual polyphenolic compounds, as described by Brown et al. [61].

## 4. Conclusions

The present study showed for the first time the evolution of the phenolic composition for seed and skin Tannat grape extracts during grape ripening. It also demonstrated that these extracts have excellent in vitro inhibitory potential against α-glucosidase. The anthocyanin and stilbene compounds do not seem to be involved in the inhibition capacity. Flavanols might be involved, as the α-glucosidase inhibition was correlated with their contents, but further research is needed to confirm this hypothesis.

## Figures and Tables

**Table 1:** Total polyphenolic content of skin and seed Tannat grapes at different ripening stages: First Stage (FS), Before Veraison (BV), Veraison (V) and Maturity (M).

Tannat Extracts		Stilbenes * (mg/kg FW)	Flavan-3-ols ** (g/kg FW)	Anthocyanins ** (g MGE/kg FW)	Total Polyphenols * (g GAE/kg FW)
FS	seeds	nd	35.24 ± 2.56 ^a^	nd	48.56 ± 1.74 ^ab^
	skins	0.81 ± 0.20 ^b^	24.13 ± 6.68 ^b^	nd	30.29 ± 0.75 ^bc^
B	seeds	nd	43.22 ± 2.59 ^a^	nd	58.44 ± 6.1 ^a^
	skins	2.81 ± 1.35 ^ab^	16.23 ± 4.93 ^b^	nd	19.43 ± 1.87 ^c^
V	seeds	nd	45.45 ± 6.48 ^a^	nd	65.29 ± 5.33 ^a^
	skins	1.08 ± 0.08 ^b^	4.34 ± 0.47 ^c^	1.42 ± 0.27 ^b^	10.33 ± 1.04 ^c^
M	seeds	nd	37.34 ± 3.36 ^a^	nd	47.87 ± 6.48 ^ab^
	skins	5.18 ± 2.04 ^a^	4.63 ± 0.19 ^c^	8.70 ± 0.75 ^a^	27.07 ± 2.92 ^bc^

* Values are means of three biological replicates. ** Values are means of three biological replicates x three technical replicates (*n* = 9). nd = not detected. Significant differences between treatments at *p* < 0.05 are noted with letters (a–c), as measured by the Tukey test. MGE: Malvidine-3-*O*-Glucoside Equivalent; GAE: Gallic Acid Equivalent; FW: Fresh Weight

**Table 2:** Quantification of individual polyphenols of skin and seed Tannat grapes at different stages of ripening. All values are the mean of three biological replicates.

Polyphenolic Compounds	Skins				Seeds			
	FS	BV	V	M	FS	BV	V	M
**Stilbenes** (mg/kg FW)								
*Cis*-resveratrol	0.007 ± 0.001	0.011 ± 0.007	0.016 ± 0.007	0.101 ± 0.028	-	-	-	-
*Trans*-resveratrol	0.039 ± 0.011	0.014 ± 0.005	0.051 ± 0.007	0.062 ± 0.011	-	-	-	-
*Trans*-piceid	0.152 ± 0.033	0.151 ± 0.070	0.221 ± 0.005	0.746 ± 0.119	-	-	-	-
*Cis*-piceid	0.357 ± 0.056	0.309 ± 0.118	0.344 ± 0.026	1.390 ± 0.479	-	-	-	-
*Cis*-piceatannol	-	-	0.011 ± 0.003	0.152 ± 0.018	-	-	-	-
*Trans*-piceatannol	-	-	0.013 ± 0.002	0.163 ± 0.017	-	-	-	-
*Trans*-astringin	0.032 ± 0.008	0.044 ± 0.009	0.163 ± 0.020	1.273 ± 0.357	-	-	-	-
*Cis*-astringin	0.064 ± 0.036	0.108 ± 0.063	0.103 ± 0.017	0.487 ± 0.166	-	-	-	-
ε-viniferin	0.102 ± 0.039	1.669 ± 0.687	0.128 ± 0.016	0.469 ± 0.038	-	-	-	-
ω-viniferin	0.005 ± 0.001	0.418 ± 0.027	0.001 ± 0.000	0.029 ± 0.007	-	-	-	-
δ-viniferin	0.004 ± 0.000	0.037 ± 0.003	0.006 ± 0.002	0.221 ± 0.011	-	-	-	-
Pallidol	0.003 ± 0.001	0.010 ± 0.009	0.003 ± 0.000	0.042 ± 0.004	-	-	-	-
Hopeaphenol	0.008 ± 0.004	0.011 ± 0.004	0.001 ± 0.000	0.011 ± 0.009	-	-	-	-
Isohopeaphenol	0.040 ± 0.004	0.024 ± 0.016	0.029 ± 0.006	0.061 ± 0.002	-	-	-	-
**Anthocyanins** (mg MGE/kg FW)								
Delphinidin-3-*O*-glu	-	-	181.85 ± 24.31	1036.81 ± 76.09	-	-	-	-
Cyanidin-3-*O*-glu	-	-	134.13 ± 18.29	317.03 ± 18.71	-	-	-	-
Petunidin-3-*O*-glu	-	-	210.89 ± 29.55	1193.13 ± 75.74	-	-	-	-
Peonidin-3-*O*-glu	-	-	185.68 ± 26.25	694.38 ± 36.33	-	-	-	-
Malvidin-3-*O*-glu	-	-	455.83 ± 83.27	3609.86 ± 240.78	-	-	-	-
Peonidin-3-*O*-acetyl-glu	-	-	50.92 ± 2.70	224.57 ± 14.07	-	-	-	-
Malvidin-3-*O*-acetyl-glu	-	-	79.91 ± 6.48	778.08 ± 62.78	-	-	-	-
Peonidin-3-*O*-coum-glu	-	-	58.49 ± 6.81	237.19 ± 16.27	-	-	-	-
Malvidin-3-*O*-coum-glu	-	-	65.67 ± 8.54	604.70 ± 39.03	-	-	-	-
**Flavan-3-ols** (mg/kg FW)								
Catechin	1539.40 ± 454.85	731.77 ± 156.40	143.71 ± 34.34	-	1942.38 ± 260.92	4620.67 ± 290.95	5462.36 ± 331.06	2892.28 ± 237.32
Epicatechin	-	-	-	-	730.15 ± 126.63	3024.21 ± 384.69	4944.27 ± 303.71	2899.74 ± 292.72
Epicatechin gallate	-	-	-	-	2777.57 ± 353.07	4438.71 ± 297.00	4796.60 ± 405.72	2642.75 ± 199.44
Catechin P	-	-	-	-	2072.75 ± 178.05	2636.23 ± 149.28	2087.75 ± 156.28	1951.14 ± 297.05
Epicatechin P	16,142.07 ± 518.50	10,257.28 ± 276.46	4203.44 ± 305.93	4625.27 ± 135.65	20,745.92 ± 1206.95	21,957.21 ± 543.21	21,907.22 ± 971.52	21,338.75 ± 1436.20
Epicatechin gallate P	-	-	-	-	6972.59 ± 352.99	6539.24 ± 105.90	6251.56 ± 730.40	5619.76 ± 353.71
Epigallocatechin P	6451.77 ± 260.83	5245.72 ± 647.76	-	-	-	-	-	-

FS = First Stage; BV = Before Veraison; V = Veraison; M = Maturity; glu = glucoside; coum = coumaroyl; P = phloroglucinol; FW = Fresh Weight.

**Table 3:** Summary of the precision results of the seed extract at maturity stage.

Added Concentration of Grape Extracts (g/L)	Abs Value (Mean ± S_R_)	CV_R_ (%)	S_RW_	CV_RW_ (%)
0	2.50 ± 0.10	3.5	0.2	8.8
0.25	1.90 ± 0.10	6.6	0.2	12.0
0.50	0.90 ± 0.10	11.5	0.2	26.1
1.00	0.30 ± 0.10	16.3	0.1	46.8

S_R_ = repeatability standard deviation; CV_R_ = repeatability coefficient of variation; S_RW_ = reproducibility standard deviation; CV_RW_ = reproducibility coefficient of variation; Abs = absorbance.

**Table 4:** Kinetic parameters of α-glucosidase inhibitory capacity by skin and seed extracts at the first stage of ripening.

[I] (mM)	K_m_ (mM/L)	V_max_ (1/min)
**Control**		
0	0.7395	0.1301
**Skin**		
0.025	9.0839	0.2657
0.05	10.0296	0.0823
0.075	7.0813	0.0283
0.1	6.8055	0.0218
**Seed**		
0.025	1.6743	0.1042
0.05	2.6553	0.0735
0.075	2.9287	0.0653
0.1	3.3618	0.0593
